# Seeing Minds in Others – Can Agents with Robotic Appearance Have Human-Like Preferences?

**DOI:** 10.1371/journal.pone.0146310

**Published:** 2016-01-08

**Authors:** Molly C. Martini, Christian A. Gonzalez, Eva Wiese

**Affiliations:** Department of Psychology, George Mason University, Fairfax, Virginia, United States of America; University of Tuebingen Medical School, GERMANY

## Abstract

Ascribing mental states to non-human agents has been shown to increase their likeability and lead to better joint-task performance in human-robot interaction (HRI). However, it is currently unclear what physical features non-human agents need to possess in order to trigger mind attribution and whether different aspects of having a mind (e.g., feeling pain, being able to move) need different levels of human-likeness before they are readily ascribed to non-human agents. The current study addresses this issue by modeling how increasing the degree of human-like appearance (on a spectrum from mechanistic to humanoid to human) changes the likelihood by which mind is attributed towards non-human agents. We also test whether different internal states (e.g., being hungry, being alive) need different degrees of humanness before they are ascribed to non-human agents. The results suggest that the relationship between physical appearance and the degree to which mind is attributed to non-human agents is best described as a two-linear model with no change in mind attribution on the spectrum from mechanistic to humanoid robot, but a significant increase in mind attribution as soon as human features are included in the image. There seems to be a qualitative difference in the perception of mindful versus mindless agents given that increasing human-like appearance alone does not increase mind attribution until a certain threshold is reached, that is: agents need to be classified as having a mind first before the addition of more human-like features significantly increases the degree to which mind is attributed to that agent.

## Introduction

Constructing artificial agents that can engage in intuitive social interactions with their human partners is not only an engineering endeavor, but one that requires a deeper understanding of how humans process and engage in interactions with non-human agents. In order to define what makes interactions intuitive, one must first understand how humans interact with each other, that is: what sort of signals they use for communication and what sort of knowledge is needed to predict and understand the behaviors of others. Psychological research has shown that when we interact with other people, we need to understand whom we are interacting with and what this person is going to do next [1]. Based on this knowledge, we make inferences about the person’s internal states (e.g., intentions, beliefs, feelings) in order to explain, understand and predict their behavior—a process that is commonly referred to as *mentalizing* [2]. Mentalizing is crucial for successful social interactions, given that they require profound understanding of how others think, feel, and act [3]. Identifying the different factors that contribute to an increased likelihood of mentalizing in interactions with non-human agents provides insight into how human observers may approach interacting with a social robot, which in turn can lead to design recommendations that may facilitate a more naturalistic interaction experience.

The current paper addresses this challenge in two ways: First, we present a theoretical framework that summarizes physical, cognitive, motivational and context variables that have been shown to increase the likelihood of attributing mental states to others (built upon the work of [3] and [4]; see Table 1). Second, we examine whether the physical appearance of an agent influences the likelihood with which mind is attributed and whether different internal states have different threshold levels before they are readily ascribed to that agent. In order to investigate this question, we created agent images of varying degrees of humanness ranging from mechanistic to humanoid and from humanoid to human and asked participants to rate these agents regarding their potential for having different internal states (e.g., feeling pain, having a sense of humor). Results from this study are used to formulate a mathematical model that determines the relationship between appearance and mind attribution and estimates appearance threshold levels for different internal states.

**Table 1:** Theoretical Framework of Factors that can Increase Mentalization of Social Robots.

	Variables		References
**Observer Features**	**Cognitive**	Agent knowledge: mind attribution, mental states	Frith & Frith [1]
			Waytz et al. [3]
		Agent behavior: intentionality, motives, cause of behavior	Dennett [5]
			Wiese et al. [6]
			Ristic & Kingstone [19]
	**Motivation**	Effectance	Epley et al. [4]
		Sociality	Ames et al. [11]
			Davis et al. [12]
			Epley et al. [13]
		Sense of control	Kay et al. [10]
**Agent Features**	**Behavior**	Predictability	Waytz et al. [3]
			Rosset [9]
		Negative consequences	Morewedge [15]
		Motion pattern	Heider & Simmel [7]
			Abell et al. [16]
			Castelli et al. [17]
			Klein et al. [18]
	**Appearance**	Similarity to humans	Kiesler [20]
			Looser & Wheatley [21]
		Design type: functional and biologically inspired	Fong, Nourbakhsh, & Dautenhahn [22]

Summarizes the work by Epley et al. [4], Waytz et al. [3], and more recent research.

### Theoretical framework on variables that facilitate mind attribution

When we try to reason about the mental states of others, we first need to decide whether an agent has a mind and, therefore is in principle, capable of having internal states [1]. This question is easy to answer for human interaction partners who are intentional agents by definition and are believed to have mental states. Machines by contrast do not possess mental states, but are believed to be physical entities with pre-programmed behaviors. Beliefs about internal states of others are generally based on experience or pre-existing knowledge we have about the world [1] and bias us to adopt specific attitudes or stances towards other agents, which are then used as mental models to explain observed agent behavior [5]: For instance, beliefs that an agent has a mind trigger the adoption of the *intentional stance*, which involves treating the agent as a rational entity with beliefs, desires and goals. The intentional stance is thought to be the best strategy to predict the behavior of agents that are truly intentional. However, if there is doubt about the intentionality of an agent’s behavior, the likelihood for adopting the intentional stance decreases, and the adoption of other stances becomes more likely. One could, for instance, adopt the *design stance* in order to explain the behavior of machines that are designed to function in certain ways (e.g., select objects in production), or the *physical stance* in order to explain the behavior of simple objects based on the laws of physics and chemistry (e.g., ball falls to the ground due to gravity).

In consequence, the same agent behavior can be explained in different ways depending on which stance is adopted towards an agent. If a human agent is observed, the intentional stance is adopted and his/ her behavior is explained based on particular internal states, such as desires, feelings, and preferences. For instance, if a human agent is looking at a red apple, one may be tempted to think that the agent is hungry and wants to eat the apple. By contrast, if the agent is believed to be a machine, one would adopt the design stance and be less tempted to think that it wants to eat the apple because it is hungry. Instead, one might speculate that the machine was programmed to look at red objects in order to select them for production purposes. Crucially, depending on the inferences we draw about the specific motives of an agent, we ascribe more or less social relevance to its actions, which has been shown to strongly influence the amount of cognitive resources we are willing to invest in the interaction [6]. Thus, agents that trigger the adoption of the design stance as opposed to the intentional stance are less likely to be treated as agents whose actions are socially relevant. As a consequence, less cognitive resources would be invested in the interaction, which could negatively impact joint action performance in human-robot interaction [6].

Given the relationship between mind attribution (i.e., assuming that an agent has a mind) and cognitive performance, it is important to investigate under which conditions humans are willing to explain the behavior of non-human agents in terms of mental states. One straightforward assumption would be that the adoption of the intentional stance is a linear function of the agent’s human-likeness: the more human-like (machine-like) an agent is, the more (less) likely it is that the intentional stance is adopted. However, the difficulty with this approach is which factors are taken into consideration when judging the human-likeness of an agent: On the one hand, artificial agents share physical features with humans (e.g., facial features, body shape, having limbs), but on the other hand they are qualitatively different from humans in terms of their capability of actually having a mind. Given that artificial agents do not truly possess minds, their behavior would be better explained as machine-like (i.e. mechanistic) than human-like (i.e. intentional). However, adopting the design stance towards artificial agents is not what people seem to do when explaining or predicting their behavior [3]. Rather, it has been shown that the same artificial agent can be treated as machine- or human-like depending on one’s beliefs about the cause of the agent’s actions. Wiese et al. [6], for instance, demonstrated that participants are more willing to follow the gaze direction of a robot under the assumption that its eye movements represent human behavior rather than pre-programmed behavior, which resulted in better overall performance in the intentional, but not in the pre-programmed condition. Thus, mind attribution seems to not only take place for agents that actually have a mind (e.g. humans), but also for agents that do not have a mind (e.g., robots). In fact, studies from the 1940s have shown that mind can even be attributed to geometrical figures that do not have human-like features at all, as long as their movement patterns resemble social interactions, for instance a big triangle chasing a small triangle [7].

In line with these observations, mind attribution seems to be a highly variable process that strongly depends on dispositional and situational factors on the observer’s side ([4], for a review) as well as the specific features of a given agent [8]. With regard to observer features, it has been shown that when uncertainty is high and no better explanation is available (e.g., lack of knowledge about computers), participants seem to revert to intentional strategies to explain behavior of artificial agents/machines [9]. Similar intentionality biases are observed when humans feel a lack of control over a situation or when being under the impression that the observed behavior might be random [10]. In addition to these cognitive factors, the motivation to understand behavior (i.e., effectance) has also been shown to have a strong influence on mind attribution [4]. For instance, if socially interacting with an agent is thought to increase the effectiveness of task execution or is assumed to take place repeatedly in the future, then behavior is more likely to be explained in intentional rather than in mechanistic terms [4]. Likewise, if motivation to engage in social interactions is high (i.e., sociality) or if the individual feels lonely but human interaction partners are not present, the behavior of non-human agents is more likely to be explained in intentional terms compared to conditions when social needs are low [11–13]. Consequently, certain individual traits (i.e., the need to belong) can be considered to be relevant observer features that influence mind attribution by increasing one’s motivation to understand observed agent behavior [14].

As for agent features, behavior and appearance seem to have the strongest influence on the degree to which mind is attributed to non-human agents. With regard to behavior, it has been shown that agents whose behavior is hard to predict trigger the need for control and appear to be more mindful than predictable agents [8]. By the same token, behavior resulting in negative consequences is more likely to be attributed to agents with minds than to agents without minds (e.g., Morewedge [15]). Heider and Simmel [7] also demonstrated that behavior of geometrical figures, which do not possess human-like appearance at all, can still be described in intentional terms as long as their motion patterns resemble social interaction scenarios [16–18]. Likewise, other studies have shown that the same stimulus can be perceived as an intentional or mechanistic agent depending on participants’ beliefs about the cause of the agent’s behavior [6]. In line with this, Ristic and Kingstone [19] showed that an ambiguous stimulus that could either be perceived as a non-social stimulus (i.e., car with wheels) or as a social stimulus (i.e., face with a hat) facilitated performance only in the social condition, but not in the non-social condition. Importantly, in both studies the perceptual features of the stimulus did not differ between conditions, but only beliefs about the stimuli varied. The studies outlined above provide evidence that agent behavior seems to influence mind attribution to non-human agents independent of the agent’s actual appearance.

However, appearance remains an influential factor for mind attribution as it has been shown that artificial agents who physically resemble human agents are more likely to trigger mind attribution [20], even if they do not show human-like behavior. For instance, research on face perception suggests that decisions on whether an agent has a mind and is alive is categorically based on holistic processing of an agent’s facial features with eyes being disproportionately informative compared to other physical features such as skin and nose [21]. Specifically, the authors found that morphs presented on a spectrum from puppet to human triggered the attribution of animacy and intentionality only after a categorical threshold biased towards the human end of the spectrum was passed (mind attribution was measured using a 7-point Likert scale).

### Aim of the Study

The studies outlined above suggest that mind attribution is a complex process that depends on dispositional and situational features on the observer’s side [4], as well as on the behavioral and physical aspects of the agent to whom the mind should be attributed. These studies also show that agents who by definition have no minds (e.g., puppets, robots) can be treated *as if* they were possessing minds under the right circumstances. More importantly, attributing mind to artificial agents has been shown to improve cognitive performance with those agents [6], which makes identifying factors that trigger mind attribution to artificial agents an important topic for cognitive psychology.

**Fig 1:**
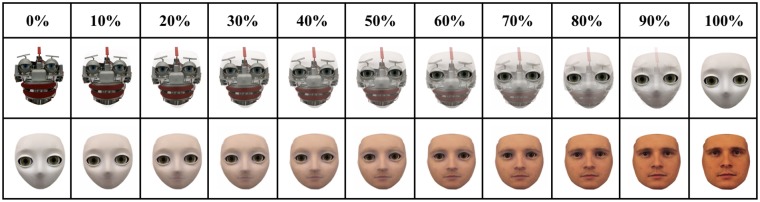
Experimental Stimuli. The morph spectrum in 10% increments ranging from 100% mechanistic (top far left) to 100% humanoid (top far right) and then from 100% humanoid (repeated bottom far left) to 100% human (bottom far right).

Considering the different ways agent appearance can be manipulated, the current study aims to better examine how physically human an agent needs to appear before intentionality is bestowed onto it (Experiment 1), and if mentalizing about different internal states (e.g., preferences, emotional states) has different appearance thresholds before humans begin to see these agents as social, intentional beings (Experiment 2). To answer these questions, we created images of agents that had different degrees of human-like appearance and asked participants to subjectively rate each morph in terms of its ability to have different internal states. The images of the agents were created by morphing in 10% increments the image of a mechanistic robot with a humanoid robot and then morphing the humanoid robot with a human image. As a result, we got a morph spectrum that ranged from 100% mechanistic robot to 100% humanoid robot and then to 100% human (see Fig 1). With this manipulation we were able to measure the relationship between the degree of physical humanness of an agent and ratings regarding its believed mental states.

The results of this study are interesting in two ways: First, the question of how physical appearance and mind attribution interact is important for cognitive psychology because it helps to understand to which degree humans take appearance into account when making judgments about another’s internal states. Second, the study provides information for social roboticists on how to design artificial agents that will reliably trigger the attribution of mental states. Given how difficult and costly it is to emulate realistic human appearance in social robots, defining the minimum level of physical humanness required to trigger the attribution of different mental states will also enable designers to more reliably determine the sufficient level of humanness a robot needs to display in order to trigger the attribution of mental states.

## Experiment 1

The goal of Experiment 1 is to determine if mind attribution is dependent on humanlike appearance and to model this potential relationship with an appropriate mathematical description. In order to address this issue, morphed agents with differing degrees of humanness were presented and rated on five items representing five different internal states. Qualtrics was used to create an online survey containing images and question items that could be linked to Amazon Mechanical Turk, an online marketplace where individuals can select to participant in various studies for small monetary rewards.

## Methods and Materials

### Participants

One hundred and sixty Amazon Mechanical Turk workers completed the 5-item per image online mental attribution survey. The survey took eleven minutes to complete and participants received $0.20 for their participation via their Mechanical Turk account. Participants were majority female (N = 94), white (N = 115), in their late-thirties (Age_avg_ = 39.07, SD_age_ = 13.60), and high school educated or above. All were reportedly native English speakers except for seven who each had 15 or more years of experience speaking English.

### Ethics Statement

The Office of Research Integrity and Assurance (ORIA) at George Mason University approved the design of this experiment and determined the project to be exempt from IRB review according to federal regulations. As the research presented no more than minimal risk of harm to the participants and the data was analyzed anonymously, a waiver of signature of informed consent was granted. While no written consent was obtained, a consent form approved by ORIA was shown at the beginning of the study informing participants of their rights. Participants had to acknowledge their consent via a response item before they could continue on to the survey.

### Materials

FantaMorph software was used to create a morphing spectrum across the three different agents using 10% increments (Fig 1). The mechanical agent represents the robot EDDIE, which was developed at the TU Munich and the humanoid agent represents the S2 humanoid head developed by Meka robotics. The image of the human agent was taken from the Karolinska Directed Emotional Faces database [23]. Each image within the mechanistic to humanoid range was presented at 248 x 259 pixels on Qualtrics while the humanoid to human images were presented at 248 x 277 pixels to account for the human head being larger in height than the other two agent images.

In order to measure the degree to which mental states were ascribed to different agents we used a questionnaire consisting of five items that asked participants to rate the social skills of a given agent on a 7-point Likert-scale (see Table 2 below). The first two questions were taken from a social identity and mind perception study by Hackel, Looser, and Van Bavel [24]. The two questions asked participants to rate how much each face looked alive (“Alive”) and how much each face looked like it had a mind (“Mind”) on a 7-point scale (1 = *Definitely not alive/Definitely has no mind* to 7 = *Definitely alive/Definitely has a mind)*. Participants were told in the instructions that a human mind differs from that of an animal or a machine, and an animal is alive while a rock is not to help clarify the meaning of these items (see [24] for a more detailed description).

**Table 2:** The Five Internal States of Experiment 1 and Their Corresponding Items.

Internal State	Item
*Alive*	Please rate how much this face looks alive.
*Mind*	Please rate how much this face looks like it has a mind.
*Feels Pain*	Do you think this agent would feel pain if it tripped and fell on hard ground?
*Hangs Out*	Do you think this agent likes to hang out with friends?
*Converses*	Do you think this agent would be an interesting conversationalist?

The remaining three items asked participants to rate if they thought the agent would feel pain if it tripped and fell on hard ground (“Feels Pain”), if the agent likes to hang out with friends (“Hangs Out”), and if the agent would be an interesting conversationalist (“Converses”). These three items were also rated on a 7-point scale (1 = *Definitely Not* and 7 = *Definitely*).

### Design and Procedure

Participants were told that they would be shown a series of faces representing different agents and that their task was to rate the perceived social skills of each agent. The presentation of the morphed images (Fig 1) was randomized and for each image participants answered the five items described above. Upon completing the agent ratings, participants filled out a demographic questionnaire and then received a code to enter on the Amazon Mechanical Turk site to collect their payment.

### Data Analysis

The data analysis process was conducted in two stages. In the first stage we attempted to identify the best fitting model of the relationship between the degree of humanness of the morph and the perceived likelihood of the morph possessing a specific internal state. In the second stage we applied the best fitting model to each specific item in order to examine threshold differences across the five internal states.

**Fig 2:**
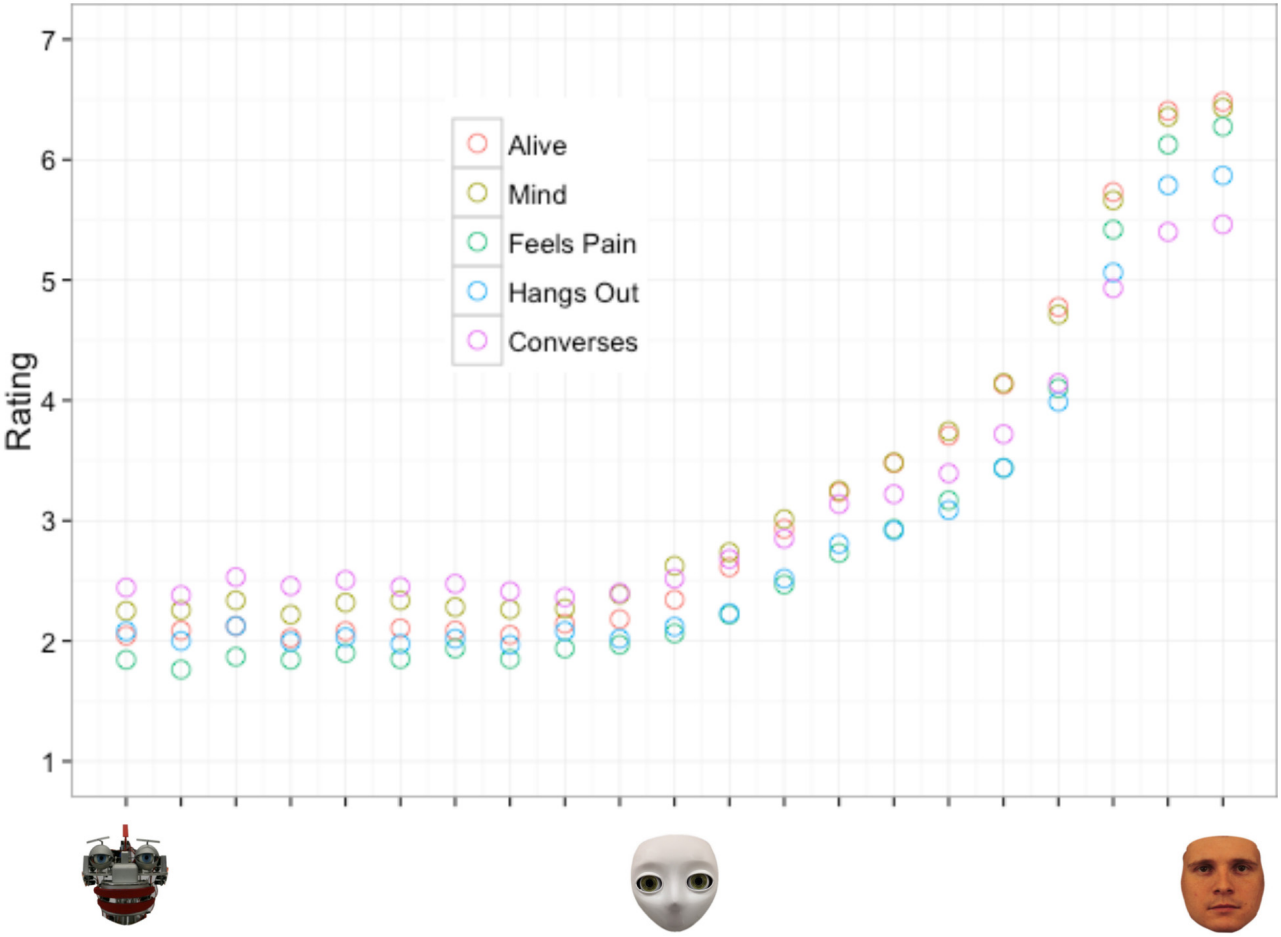
Experiment 1 scatterplot of average ratings of perceived intentionality by degree of humanness for the five different internal states. Each increment on the x-axis is equivalent to a 10% increase along the mechanistic to humanoid to human spectrum.

In order to narrow the potential models to test, we first plotted mean ratings of perceived likelihood of the morph possessing each internal state by the degree of humanness of the morph (See S1 Data for the raw data of Experiment 1). Fig 2 depicts this relationship to be nonlinear as increases in human-likeness yielded negligible increases in perceptions of intentionality along the morphs between mechanistic and humanoid agents, but yielded large increases in perceived intentionality along the morphs between the humanoid and human agents. Based on this observed non-monotonic relationship we considered three different potential models: 1) a quadratic model 2) an exponential model and 3) a two-linear model.

A quadratic model defines the relationship between an independent variable, *X*, and an outcome variable, *Y*, as a function of the linear and squared components of *X* as well as a constant or intercept term. In formula 1 below, *a* represents the constant parameter, *b* represents the linear parameter and *c* represents the squared parameter.

$$Y\sim a+bX+cX^{2}$$ (1)

An exponential model defines the relationship between an independent variable, *X*, and an outcome variable, *Y*, as the product between a constant term and base raised to the *X* power. In formula 2 below, *a* represents the constant parameter and *b* represents the base parameter.

$$Y\sim ab^{X}$$ (2)

Lastly, a two-linear model defines the relationship between an independent variable, *X*, and an outcome variable, *Y*, and results in two separate linear relationships each with their respective linear and constant terms. In formula 3 below, *a* represents the constant term of line 1, *b* represents the slope of line 1, *e* represents the constant term of line 2, *c* represents the slope of line 2 and *d* represents the break point between line 1 and line 2.

$$\begin{array}{l} \text{IF}\\ X<d\\ Y\sim a+bX\\ \text{ELSE}\\ Y\sim e+cX \end{array}$$ (3)

#### Model fitting

**Fig 3:**
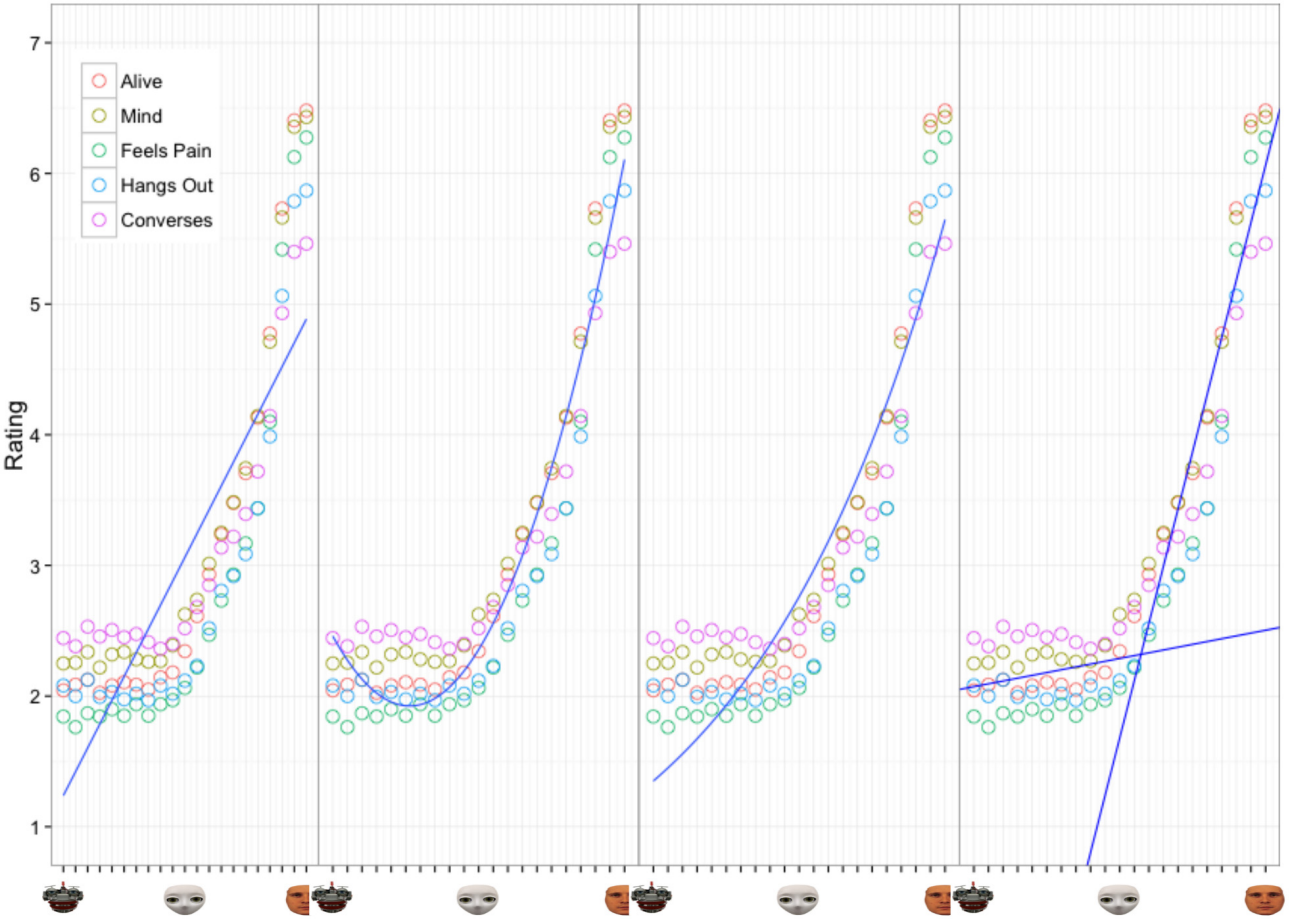
Experiment 1 Model Fits. Results of the four different model fits of the relationship between average ratings of perceived intentionality by degree of humanness for the five different internal states.

Each of the three models described above was fit with nonlinear least-squares implemented in the *easynls* R package [25]. We set the average ratings of perceived intentionally as the outcome variable and degree of humanness as reflected by the morph number as the single independent variable. In order to determine the best fitting model we computed multiple fit measures for each model: adjusted *R*^*2*^ and Akiake Information Criterion (AIC) (Table 3 and Fig 3). Adjusted *R*^*2*^ provides an estimate of the proportion of the variance in the outcome variable explained by a given model while AIC represents the relative amount of information lost given a particular model. AIC is often used in model selection because it accounts for potential overfitting by complex models with many parameters [26]. In both cases, lower AIC indicates a better model fit.

**Table 3:** The Akiake Information Criterion (AIC) and Adjusted *R*^*2*^ Values for Each Model Providing a Measure of Fit for Experiment 1.

	AIC	Adj. R^2^
Linear	232.51	0.70
Quadratic	74.14	0.93
Exponential	170.28	0.83
Two-Linear	73.77	0.94

**Fig 4:**
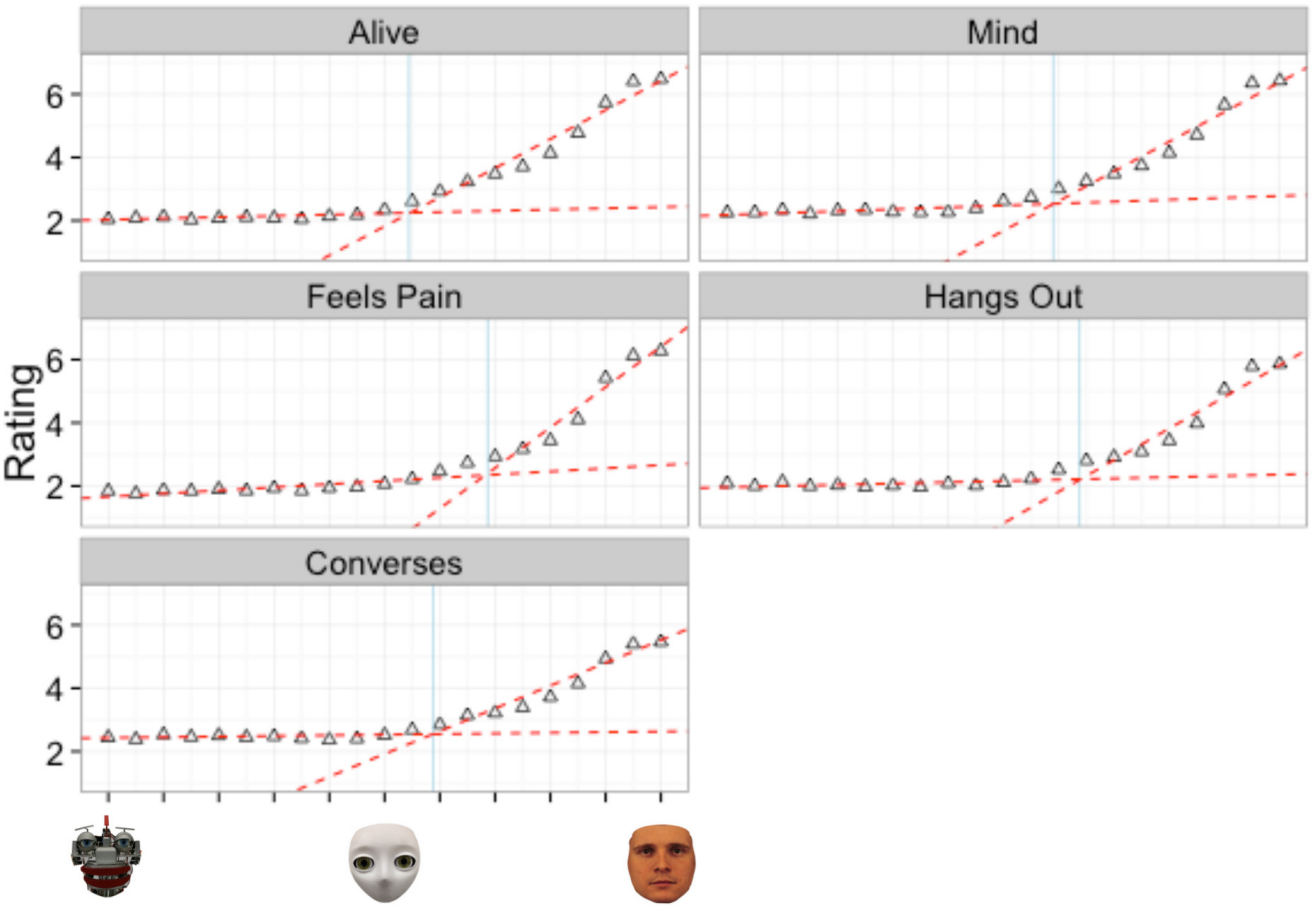
Two-linear model applied to the average ratings for each question of Experiment 1. The light blue vertical line shows where the breakpoint occurs between the two linear functions.

Once we determined that a two-linear model provided the best overall fit to our data we applied the model to the ratings of each of the five internal states (Fig 4). Specifically, we were interested in determining if there were significant differences in the breakpoint (*parameter d*) between internal states, which would suggest that the distinction between “having a mind” and “not having a mind” is in part dependent on the internal state being judged. In addition, significant differences in the average rating of intentionality for the fully mechanistic morph (*parameter a*) would suggest baseline differences in perceptions of intentionality depending on which internal state is judged. Significant differences in the slope of line 1 for mechanistic to humanoid morphs (*parameter b*) would suggest differences in the effect additional human characteristics, specifically a shell covering the wiring of the robot and creating an oval face structure, have on perceptions of intentionality between different internal states. Finally, significant differences in the slope of line 2 for humanoid to human morphs (*parameter c*) would suggest differences in the effect of physical humanness on perceptions of intentionality between internal states. As we were not concerned with parameter e, the constant for line 2 and baseline differences in perceptions of intentionality for the humanoid agent, it was excluded from further analysis. Refer to Table 4 below for the definitions of the two linear parameters.

**Table 4:** Description of the Five Parameters Estimated from a Two-Linear Model.

Parameter	Description	Interpretation
a	Intercept for Line 1	Value of internal state rating for fully mechanistic agents
b	Slope of Line 1	Increase in ratings (slope) of each internal state before breakpoint
c	Slope of Line 2	Increase in ratings (slope) of each internal state after breakpoint
d	Breakpoint of Line 1 and 2	Morph value which separates Line 1 from Line 2
e	Intercept for Line 2	Constant term for Line 2

Parameter *e*, the y-intercept of line 2 was not estimated nor of interest in our analyses.

#### Internal state comparisons

**Fig 5:**
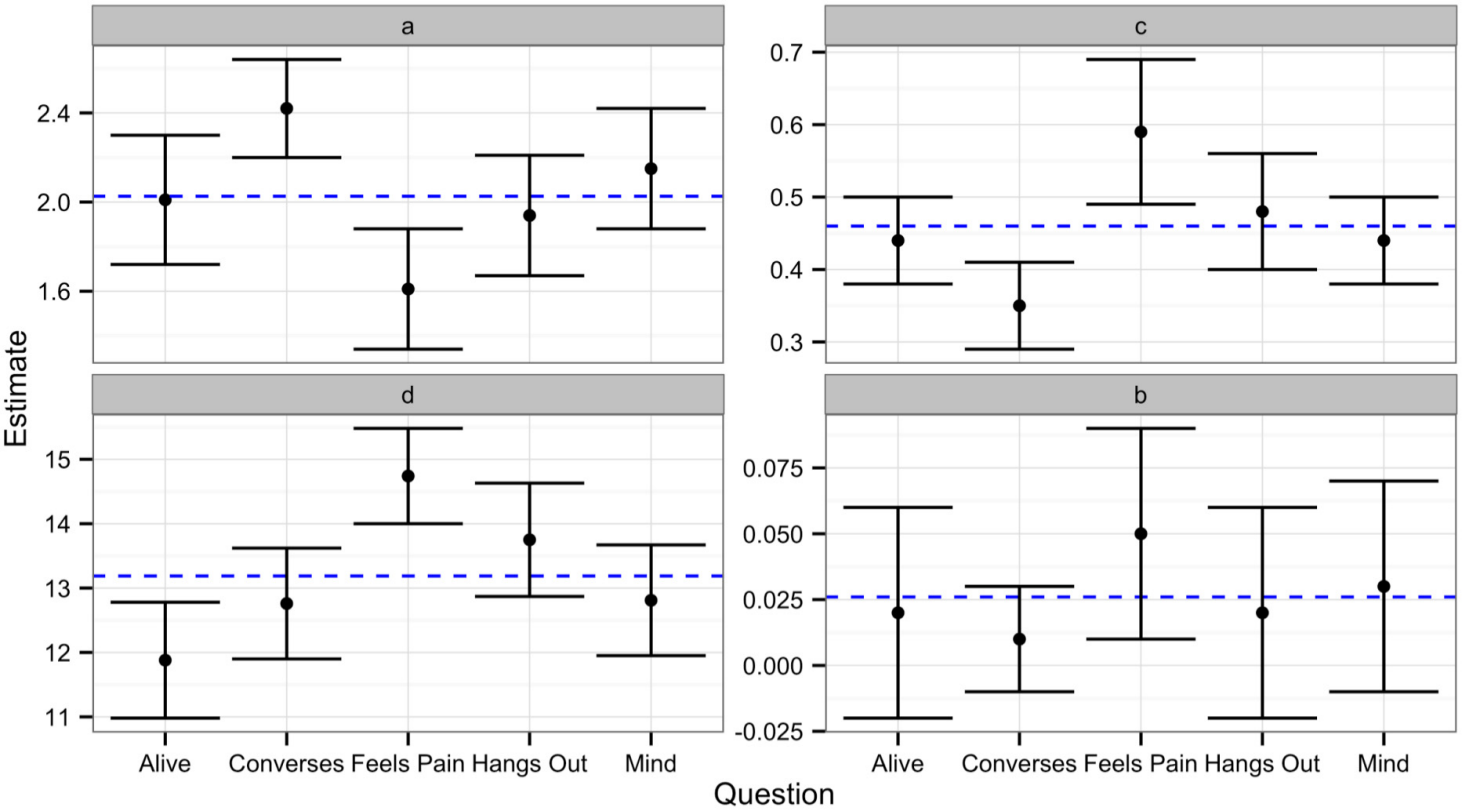
95% confidence intervals of the two-linear model parameters for each internal state of Experiment 1. Blue line is the grand mean of estimates of each parameter. Parameter a is the y-intercept of line 1, parameter b is the slope of line 1, parameter c is the slope of line 2 and parameter d is the breakpoint separating line 1 and line 2.

Rather than comparing each internal state to every other internal state per each parameter (which would have resulted in 60 separate comparisons), we compared the 95% confidence intervals of each parameter estimate to the grand mean of the average parameter estimates for each internal state. A parameter was considered significantly different from the grand mean if a 95% confidence interval around the observed parameter did not include the grand mean. In comparison, if the 95% confidence interval crossed the grand mean or overlapped with the 95% confidence interval of another parameter, then those parameters were not considered significantly different. Table 5 contains parameter estimates, standard errors and confidence intervals for each internal state. Fig 5 depicts the 95% confidence intervals of each parameter estimate to the grand mean of the parameter estimates for each internal state.

**Table 5:** Two-Linear Model Results from Experiment 1.

Parameter	Question	Estimate	SE	2.50%	97.50%
**a**	Alive	2.01	0.15	1.72	2.30
	Mind	2.15	0.14	1.88	2.42
	Feels Pain	1.61	0.14	1.34	1.88
	Hangs Out	1.94	0.14	1.67	2.21
	Converses	2.42	0.11	2.20	2.64
**b**	Alive	0.02	0.02	-0.02	0.06
	Mind	0.03	0.02	-0.01	0.07
	Feels Pain	0.05	0.02	0.01	0.09
	Hangs Out	0.02	0.02	-0.02	0.06
	Converses	0.01	0.01	-0.01	0.03
**c**	Alive	0.44	0.03	0.38	0.50
	Mind	0.44	0.03	0.38	0.50
	Feels Pain	0.59	0.05	0.49	0.69
	Hangs Out	0.48	0.04	0.40	0.56
	Converses	0.35	0.03	0.29	0.41
**d**	Alive	11.88	0.46	10.98	12.78
	Mind	12.81	0.44	11.95	13.67
	Feels Pain	14.74	0.38	14.00	15.48
	Hangs Out	13.75	0.45	12.87	14.63
	Converses	12.76	0.44	11.90	13.62

Estimate is maximum-likelihood estimate, SE is the standard error of the estimate, 2.5% and 97.5% indicates the lower and upper bounds of a 95% confidence interval around the estimate. Parameter *a* is the y-intercept of Line 1, parameter *b* is the slope of Line 1, parameter *c* is the slope of Line 2, parameter *d* is the breakpoint separating Line 1 and 2.

Our results revealed no significant differences in *parameter d*, as exhibited by the overlapping confidence intervals in Fig 5 and Table 5. This suggests that regardless of the internal state being considered, the breakpoint between mechanistic to humanoid morphs and humanoid to human morphs remains the same and is a qualitative, not quantitative difference. In addition, our results revealed no significant differences in *parameter b*. This suggests that regardless of the internal state being considered, the relationship between degree of humanness and perceived intentionality of mechanistic to humanoid morphs remains the same.

However, as indicated in Table 5 and Fig 5, we found significant differences on *parameter a*, suggesting that baseline differences in intentionality ratings may exist between different internal states (i.e., the same agent gets lower ratings on “feeling pain” compared to “having a mind”). Specifically, the ratings for conversations skills of the mechanistic agent were significantly higher than ratings regarding the other four categories (as compared to grand mean of parameter a). Our results also revealed significant differences in *parameter c*, suggesting that the degree of humanness has differential effects on the ratings of different internal states (i.e., slope for “feeling pain” is steeper than slope for “having a mind”). Specifically, significantly higher estimates of *parameter c* were observed for the relationship between degree of humanness and perceptions of feeling pain as compared to ratings of other internal rates (i.e., a one unit increase in humanness results in a greater increase in perceptions of feeling pain compared to the other internal states). Conversely, we observed a significantly lower estimate of *parameter c* for the relationship between degree of humanness and perceptions of conversation skills (i.e., a one unit increase in humanness results in a smaller increase in perceptions of conversation skills compared to the other internal states).

### Discussion

In accordance with previous studies (e.g., Hackel et al. [24]), the results of Experiment 1 suggest that the degree of human appearance of an agent is related to the degree to which mental states are attributed to that agent. However, in addition to previous studies, our findings revealed that this relationship is not linear: Increases in physical humanness from mechanistic to humanoid agents yielded only negligible increases in mind attribution, while increases in humanness from humanoid to human agents yielded large increases in mind attribution. After considering three different nonlinear models of this relationship, a two-linear model seems to fit the data best, which represents the relationship between degree of humanness and mind attribution as arising from two linear relationships separated by a breakpoint. The fact that the two-linear model fits the data best suggests a qualitative difference between mindful and mindless agents. The location of this breakpoint can be used to estimate how much human-likeness an agent needs before adding more humanness to its appearance results in significant changes of mind attribution. In Experiment 1, the breakpoint was located at around 25% humanness and there were no differences between different internal states regarding the location of this breakpoint on the spectrum. This finding implies that when judging an agent regarding a particular internal state, it does not seem to matter which internal state is evaluated, but more whether or not we think that the agent is in general capable of having a mind. The tendency to answer this question with “yes” seems to increase significantly after the breakpoint is passed and does not seem to depend on which internal state is evaluated.

However, one limitation of Experiment 1 could be that each internal state was operationalized only by one item (e.g., Having emotions → “feeling pain”), which may have resulted in a lack of sensitivity in our measurements and ultimately be responsible for not being able to detect breakpoints across different internal states. In addition, using a single broad category, e.g. “Do you think this agent has a mind?”, may have produced a higher degree of variability in average ratings by failing to provide examples of specific behaviors which a mindful agent might engage in, such as “Do you think this agent can learn?”. Finally, the small number of internal states participants responded to may not have been sufficiently diverse in order to detect differences in their breakpoints. Thus in order to validate the results of Experiment 1, we conducted a second experiment to explore the potential differences between a greater number of internal states captured by multiple items.

## Experiment 2

Experiment 2 was conducted to further expand upon the findings of Experiment 1 by examining whether the effects of physical appearance on mind attribution can be generalized to different categories of internal states (e.g., agency, animacy, emotions, goals). Participants were shown a subset of the morphed images of Experiment 1 and asked to rate the degree to which they believed a given agent possessed a given internal state. However, instead of answering five questions per image, participants were now asked to respond to 39 items per image categorized under eight different mental states in order to increase the specificity of the model. If the pattern observed in Experiment 1 persisted for an increased number and wider range of internal states, it would provide further evidence that the relationship between humanlike appearance and perceived ratings of intentionality follows the general pattern of a two-linear model. More importantly, it would support the view that intentionality is only conferred onto a robotic agent if its physical design contains some degree of humanlike appearance.

## Methods and Materials

### Participants

One hundred and fifty-eight Amazon Mechanical Turk workers completed the new 39-item per image online mental attribution survey. The average completion time was 44 minutes and participants received $0.50 for their participation via their Mechanical Turk account. Participants were majority female (N = 85), white (N = 115), in their mid-thirties (*Age*_*avg*_ = 35.75, *SD*_age_ = 12.44), and high school educated or above. All were reportedly native English speakers except for seven who each had 13 years or more of experience speaking English.

### Ethics Statement

Similar to Experiment 1, the Office of Research Integrity and Assurance (ORIA) at George Mason University approved the design of this experiment and determined the project to be exempt from IRB review according to federal regulations. As the research presented no more than minimal risk of harm to the participants and the data was analyzed anonymously, a waiver of signature of informed consent was granted. While no written consent was obtained, a consent form approved by ORIA was again shown at the beginning of the study informing participants of their rights. Participants had to acknowledge their consent via a response item before they could continue on to the survey.

### Materials

The same images used in Experiment 1 were used for Experiment 2. However, in Experiment 2 the number of morph images was reduced to eleven, starting with the mechanistic robot moving along the morph spectrum at increments of 20% (e.g., 100% mechanistic, 80% mechanistic, …, 100% humanoid, 80% humanoid, …, 100% human). There are two main reasons why the number of images was reduced: First, there were no large deviations in participant ratings across the spectrum of morphs in Experiment 1 that would not be captured by a reduced range. Second, increasing the number of questions to 39 required a reduction in number of images to keep the survey at an overall reasonable length. Qualtrics was used to create the survey, which was presented via Amazon Mechanical Turk.

In Experiment 2, we asked participants to assess the mental states of the depicted agents on eight different categories: *Agency*, *Animacy*, *Theory of Mind*, *Emotions*, *Goals and Preferences*, *Cognitive Skills*, *Social Interactions/Communicative Skills*, and *Sense of Humor*. Each category had five items except for Goals and Preferences which only had four items, and all questions were answered on a seven-point scale with 1 = *Definitely Not* to 7 = *Definitely* (see Table 6 for example items of each category; see S1 Text for all items).

**Table 6:** The Eight Mental State Categories of Experiment 2 with Example Items.

Mental State Category	Description	Item Examples
*Agency*	Ability to act independently	Do you think this agent is aware of its actions?
		Do you think this agent can make its own decisions?
*Animacy*	Alive and consciously aware	Do you think this agent looks alive?
		Do you think this agent has a mind?
*Theory of Mind*	Ability to understand the minds of others	Do you think this agent can understand your intentions?
		Do you think this agent can understand your emotions?
*Emotions*	Ability to feel physically and emotionally	Do you think this agent can experience emotion?
		Do you think this agent can experience happiness?
*Goals and Preferences*	Ability to have its own goals and preferences	Do you think this agent has desires?
		Do you think this agent has goals?
*Cognitive Skills*	Ability to learn, have thoughts, and be capable of complex behavior	Do you think this agent is capable of complex behavior?
		Do you think this agent can learn?
*Social Interactions/Communicative Skills*	Ability and willingness to socialize with others	Do you think this agent would hang out with friends?
		Do you think this agent is capable of taking care of another being?
*Sense of Humor*	Ability to understand and provide humor	Do you think this agent would understand humor?
		Do you think this agent would like jokes?

While two example items are shown, in the actual survey each category had four to five items and were rated on a 1–7 scale with 1 = *Definitely Not* and 7 = *Definitely*.

### Design and Procedure

The procedure for Experiment 2 was similar to Experiment 1: Participants were told that they would be shown a series of faces representing different agents and that each image would appear with a question asking them to rate the agent’s social skills. A total of 39 items, organized into eight different categories, was presented for each agent. Question and image pairing along with image order was randomized throughout the survey. Upon completing agent ratings, participants filled out a questionnaire assessing their demographic data and then received a code to enter on the Amazon Mechanical Turk site to collect their payment.

### Data Analysis

Data analysis was similar to Experiment 2. First, we collapsed the data into the eight different categories by averaging the items within each category. Then the data analysis process was conducted in two stages. In the first stage we attempted to identify the overall best fitting model of the relationship between the degree of humanness of the morph and the perceived likelihood of the morph possessing a specific internal state. In the second stage we applied the best fitting model to each specific item in order to examine differences across five different internal states.

#### Model fitting

**Fig 6:**
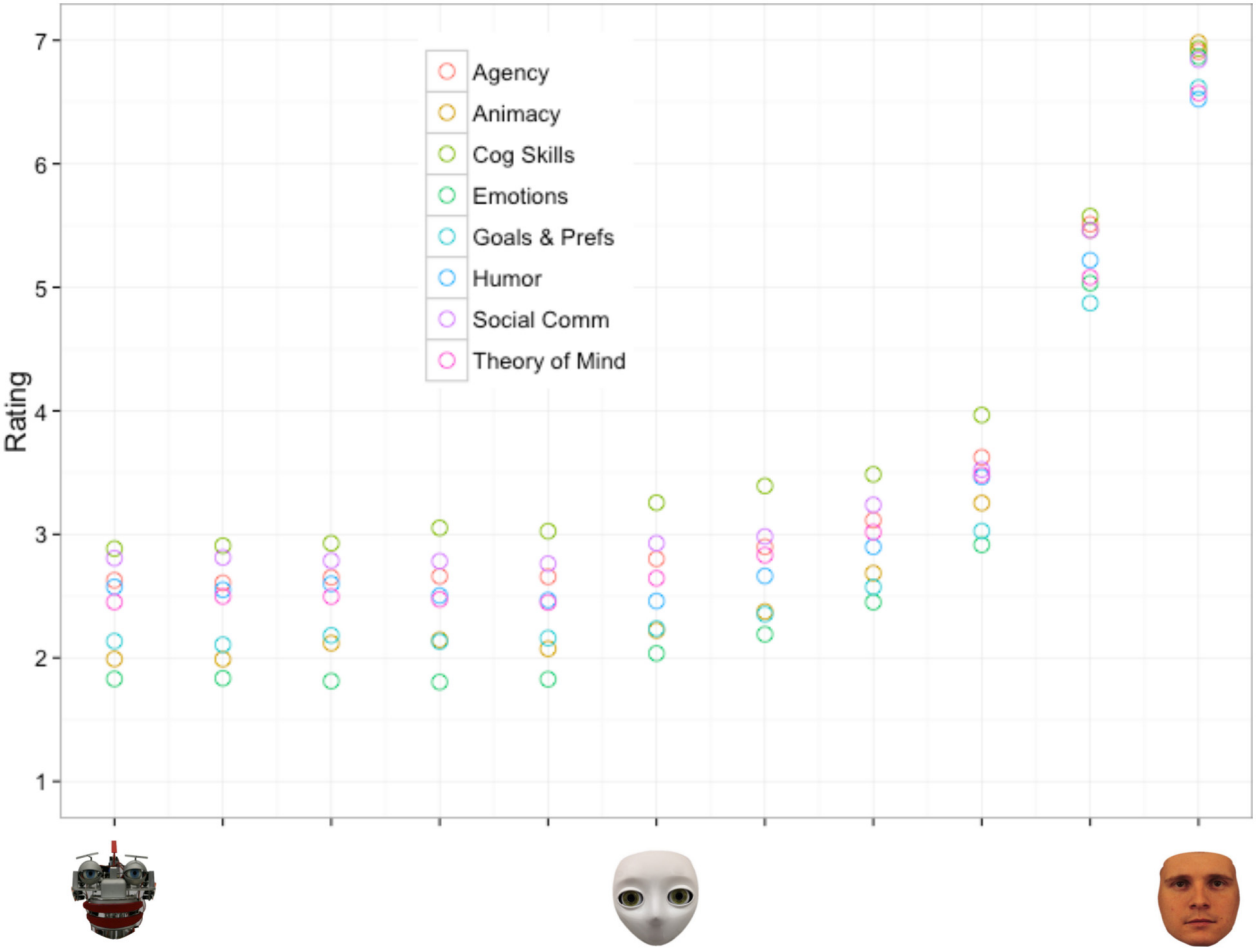
Experiment 2 scatterplot of the average ratings of perceived intentionality by degree of humanness for the eight different internal state categories.

In order to narrow the choices of potential models to test, we again first plotted the mean ratings of perceived likelihood of the morph possessing each internal state by the degree of humanness of the morph (See S2 Data for the raw data of Experiment 2). Similar to Fig 2 in Experiment 1, Fig 6 depicts a nonlinear relationship between ratings of perceived likelihood of the morph possessing a particular internal state and the degree of its physical humanness: Increases in humanness yielded only negligible increases in perceptions of intentionality along the morphs between the mechanistic and humanoid agents, but yielded large increases in perceived intentionality for the morphs between the humanoid and human agents. Based on this observed non-monotonic relationship we considered the same three models as Experiment 1: 1) a quadratic model 2) an exponential model and 3) a two-linear model.

**Fig 7:**
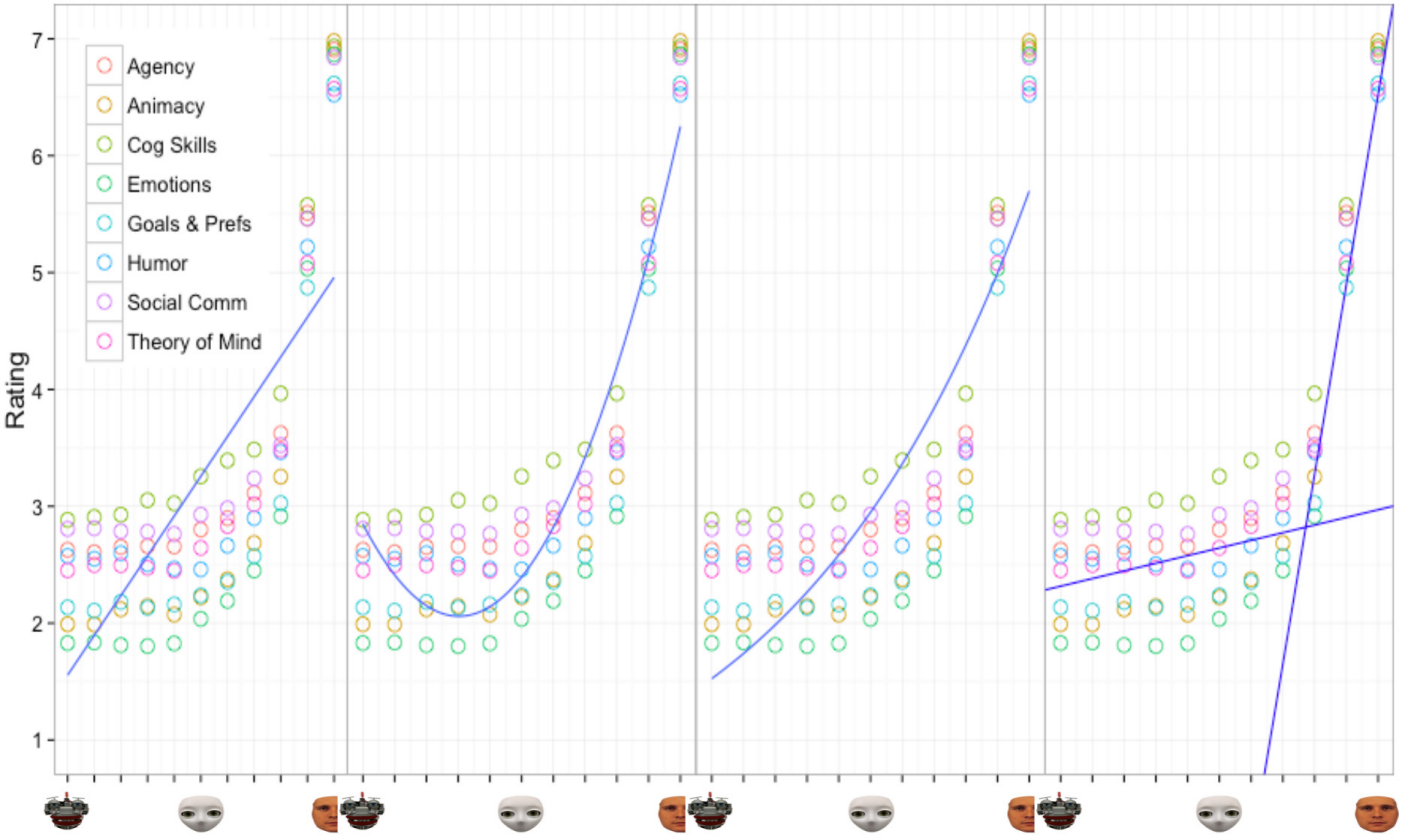
Experiment 2 Model Fits. Results of the four different model fits of the relationship between average ratings of perceived intentionality by degree of humanness for the eight different internal state categories.

Each of the three models described above was fitted and evaluated in the same manner as in Experiment 1 (see Table 7 and Fig 7 for the results of the model testing procedure). Overall, the results suggest the two-linear model fits the data best. The two-linear model yielded the highest adjusted *R*^*2*^ value and lowest AIC, suggesting that the relationship between perceived intentionality and degree of humanness is best captured by two separate linear models. Similar to Experiment 1, the two-linear model suggests that a qualitative difference exists between agents with a mechanistic appearance and those with a humanoid to human appearance. The *parameter d* in particular defines the breakpoint value where this distinction lies. Results found this breakpoint to occur between the 40% human and 60% human morphs, suggesting that on average an agent needs to have at least 50% human appearance before further increases in its degree of humanness has meaningful impact on perceptions of intentionality. It is important to note that this model stands in contrast to a continuous linear prediction whereby increases in humanness yield monotonic increases perceptions of intentionality.

**Table 7:** The Akiake Information Criterion (AIC) and adjusted *R*^*2*^ values for each model providing a measure of fit for Experiment 2.

	AIC	Adj. R^2^
Linear	241.20	0.57
Quadratic	142.01	0.86
Exponential	207.18	0.71
Two-Linear	74.33	0.94

**Fig 8:**
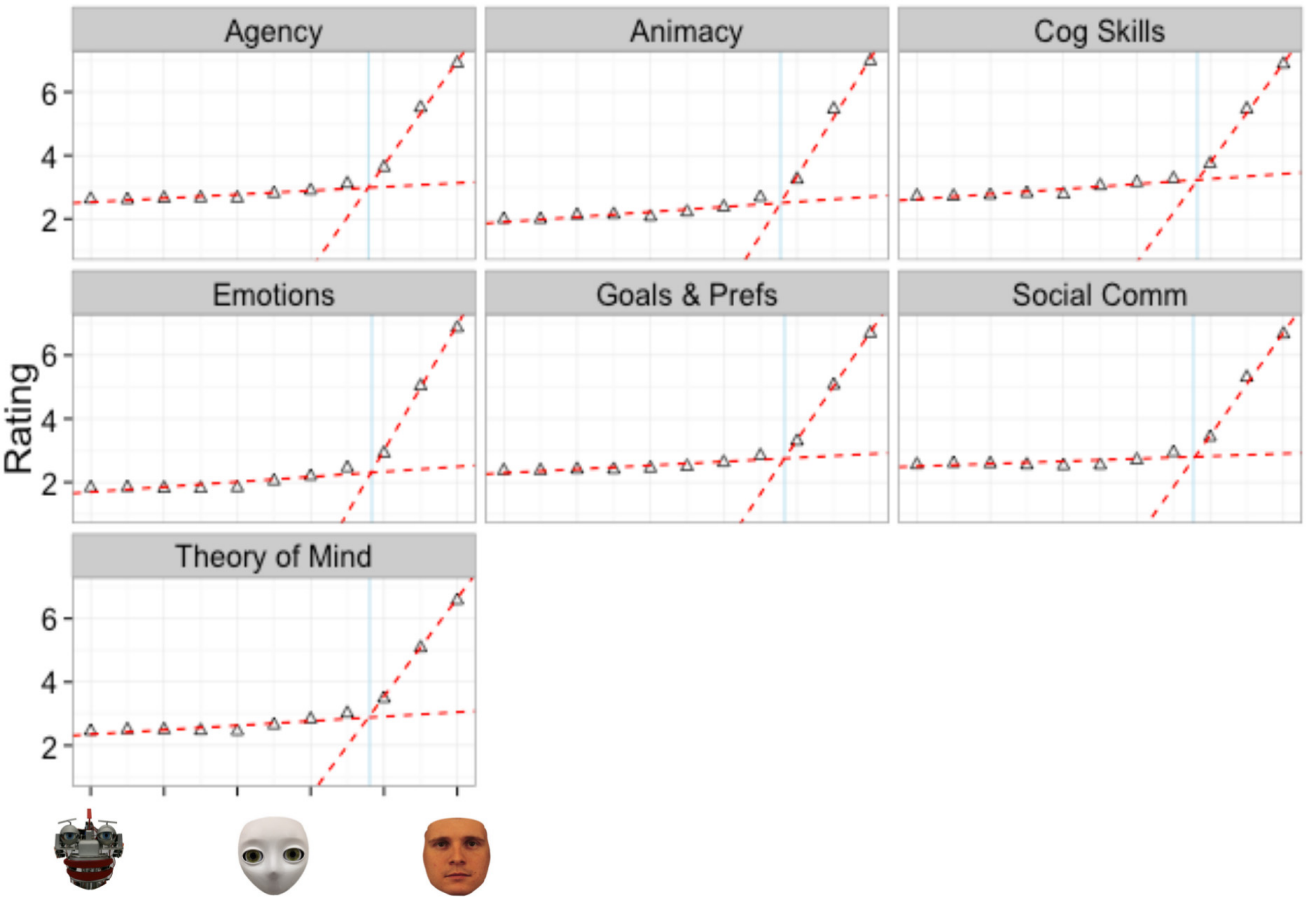
Two-linear model applied to the average ratings for each question category of Experiment 2. The light blue vertical line shows where the breakpoint occurs between the two linear functions.

Once we determined that a two-linear model provided the best overall fit to our data we applied the model to each of the eight internal states (Fig 8). Similar to Experiment 1, we were interested in identifying differences in the parameter estimates across each of the different internal states. As a reminder, the two-linear model provides estimates for four parameters, the slope (a) and intercept (b) for line one, the slope (c) for line two and the break point between the two (d).

#### Internal state comparisons

**Fig 9:**
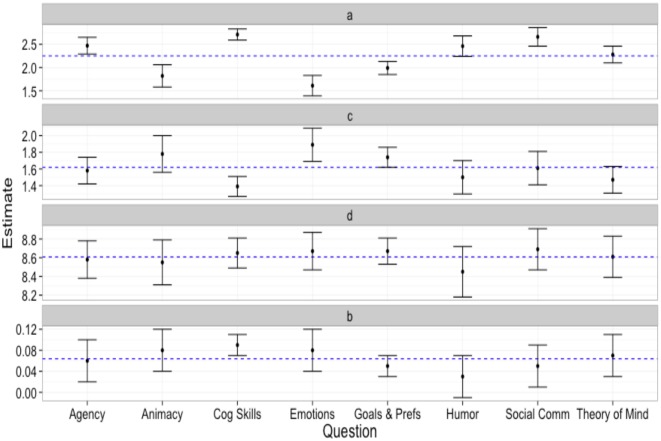
95% Confidence intervals of the two-linear model parameters for each internal state of Experiment 2. Blue line is the grand mean of estimates of each parameter. Parameter a is the y-intercept of line 1, parameter b is the slope of line 1, parameter c is the slope of line 2 and parameter d is the breakpoint separating line 1 and line 2.

We compared parameters across internal states using the same procedure as in Experiment 1: the 95% confidence intervals of each parameter estimate were compared to the grand mean of the parameter estimates for each internal state. Table 8 contains parameter estimates, standard errors and confidence intervals for each internal state. Fig 9 depicts the 95% confidence intervals of each parameter estimate to the grand mean of the parameter estimates for each internal state.

**Table 8:** Two-Linear Model Results from Experiment 2.

Parameter	Question Category	Estimate	SE	2.50%	97.50%
***a***	Agency	2.47	0.09	2.29	2.65
	Animacy	1.82	0.12	1.58	2.06
	Cognitive Skills	2.71	0.06	2.59	2.83
	Emotions	1.61	0.11	1.39	1.83
	Goals & Preferences	1.99	0.07	1.85	2.13
	Humor	2.46	0.11	2.24	2.68
	Social Communication	2.66	0.1	2.46	2.86
	Theory of Mind	2.28	0.09	2.10	2.46
***b***	Agency	0.06	0.02	0.02	0.10
	Animacy	0.08	0.02	0.04	0.12
	Cognitive Skills	0.09	0.01	0.07	0.11
	Emotions	0.08	0.02	0.04	0.12
	Goals & Preferences	0.05	0.01	0.03	0.07
	Humor	0.03	0.02	-0.01	0.07
	Social Communication	0.05	0.02	0.01	0.09
	Theory of Mind	0.07	0.02	0.03	0.11
***c***	Agency	1.58	0.08	1.42	1.74
	Animacy	1.78	0.11	1.56	2.00
	Cognitive Skills	1.39	0.06	1.27	1.51
	Emotions	1.89	0.1	1.69	2.09
	Goals & Preferences	1.74	0.06	1.62	1.86
	Humor	1.5	0.1	1.30	1.70
	Social Communication	1.61	0.1	1.41	1.81
	Theory of Mind	1.47	0.08	1.31	1.63
***d***	Agency	8.58	0.1	8.38	8.78
	Animacy	8.55	0.12	8.31	8.79
	Cognitive Skills	8.65	0.08	8.49	8.81
	Emotions	8.67	0.1	8.47	8.87
	Goals & Preferences	8.67	0.07	8.53	8.81
	Humor	8.45	0.14	8.18	8.72
	Social Communication	8.69	0.11	8.47	8.91
	Theory of Mind	8.61	0.11	8.39	8.83

Estimate is maximum-likelihood estimate, SE is the standard error of the estimate, 2.5% and 97.5% indicates the lower and upper bounds of a 95% confidence interval around the estimate. Parameter *a* is the y-intercept of Line 1, parameter *b* is the slope of Line 1, parameter *c* is the slope of Line 2, parameter *d* is the breakpoint separating Line 1 and 2.

The results revealed no significant differences in *parameter d*, as exhibited by the overlapping confidence intervals in Fig 9 and Table 8. This suggests that regardless of the internal state being considered, the breakpoint defining the qualitative difference between mechanistic to humanoid morphs and humanoid to human morphs remains the same. In addition, the results revealed no significant differences in *parameter b*. This suggests that regardless of the internal state being considered, the relationship between the degree of humanness and the perceived intentionality remains the same on a spectrum from mechanistic to humanoid.

However, the results did reveal significant differences in *parameter a*, suggesting baseline differences in ratings of agency, cognitive skills, animacy and emotions. Specifically, participants rated the mechanistic agent as having higher levels of agency and cognitive skills compared to the grand mean. Conversely, participants rated the mechanistic agent as having lower levels of animacy and emotions compared to the grand mean. The results also revealed significant differences in *parameter c*, suggesting that differences in the relationship between the degree of humanness and perceived intentionality may exist on the spectrum from humanoid to human. First, a significantly higher estimate of parameter c was observed for the relationship between degree of humanness and perceptions of agent emotions. This suggests that a one unit increase in humanness results in a greater increase in perceptions of emotions compared to other internal states. Second, a significantly lower estimate of parameter c was observed for the relationship between degree of humanness and perceptions of cognitive skills. This suggests that a one unit increase in humanness results in a smaller increase in perceptions of cognitive skills compared to other internal states.

### Discussion

The results of Experiment 2 replicated the results of Experiment 1 by suggesting that the degree of human-like appearance of an agent is related to mind attribution of that agent. This relationship, however, is not linear: Increases in humanness from mechanistic to humanoid agents yielded only negligible increases in mind attribution, while increases in humanness from humanoid to human agents yielded large increases in mind attribution. The results suggest that a two-linear model fits the data best, which can be interpreted as a qualitative difference between mindful and mindless agents. In contrast to Experiment 1, however, the location of the breakpoint at which the qualitative switch occurred was found at 50% humanness. The analysis of the location of this breakpoint across the eight different internal categories revealed no significant differences, which suggests that regardless of the internal state being evaluated, agents need to be roughly 50% human-like in appearance before increases in humanness yield measurable impacts on mind attribution.

One possible explanation for this effect might be that using multiple specific behavioral items related to each internal state may have increased the threshold by which an agent is considered mindful. With more specificity added to the items participants may have used a stricter criteria to evaluate the different images raising the appearance threshold level. Additionally, participants may be using stricter criteria simply because the image set is reduced, giving them less chances to compare across images in comparison to Experiment 1. It should also be noted that these findings are based on treating the morph spectrum as a continuous function from fully mechanistic to fully human; however, we did not in fact morph a fully mechanistic agent with a human. Instead the humanoid agent was used as the intermediary between the two as morphing the mechanistic agent with a human agent would not retain sufficient humanlike characteristics in the intermediate morphs to be perceived as an agent at all. Thus the two-linear model we observed may be in response to the trichotomy of the morphs used rather than capturing a true distinction between mindless and mindful agents.

## General Discussion

Attributing mental states to other agents has been shown to result in better cognitive performance in interactions with these agents [6] and depends on dispositional and situational features of the observer [3, 4, 14, 19] and on the features of the agent [16, 17, 20, 21]. In the past, a lot of effort has been put into the development of social artificial agents by providing them with reasoning systems that are based on human cognitive and developmental models [27, 28], as well as by investigating cognitive and motivational factors on the observer side that increase the likelihood that human-likeness is ascribed to the agent [4]. Only very few studies, however, have investigated the relationship between physical appearance and mind attribution [22, 24] or how mind attribution influences cognitive performance [6]. The current study addressed this issue, by i) introducing a theoretical framework of the different variables that affect mind attribution to artificial agents looking at both observer and agent characteristics, ii) by identifying the minimal level of humanlike appearance required for a mechanical being to be perceived as a mindful agent and by iii) testing whether different levels of humanlike appearance are required for different internal states to be attributed to an artificial agent.

Our results indicate that the degree of human-like appearance has a direct relationship with perceptions of intentionality and different internal states. Specifically, Experiment 1 and 2 highlight the fact that physical manipulation of agent appearance can indeed induce differing levels of mind attribution in human observers: The more human-like the appearance, the more likely it is that mental states are attributed to the agent. However, this relationship is not rectilinear nor quadratic or exponential (see Figs 3 and 7). Instead, our results suggest a two-linear relationship whereby changes in physical appearance have little effect on mind attribution until a specific breakpoint, then perceptions increase substantially as images approach 100% human appearance. For both Experiment 1 and 2, regardless of the internal state being perceived, this particular breakpoint did not occur until the humanoid to human side of the spectrum was reached.

Given these results, we propose the theory that mentalizing about robotic agents based on appearance is a two-step process in which two different mechanisms are enacted. First, a higher-level thought process is engaged in which an agent is judged on its general capability of possessing a mind. This initial judgment phase corresponds to a qualitative threshold (observed in both experiments) below which increases in appearance do not have any effect. Then once this threshold is past, a second processing phase occurs where judgments are made about particular mental states (e.g., feeling pain versus having a good sense or humor). It is in this secondary phase where mentalizing on specific traits becomes quantitatively influenced by appearance. For instance, increasing human-like appearance past the threshold resulted in higher ratings of an agent’s ability to feel pain, but lower ratings of an agent’s perceived cognitive skills. This two-step processing theory is in line with Wykowska, Wiese, Prosser, and Müller’s [29] proposed hypothesis that humans must first decide to take either an intentional or design stance [5] towards a robotic partner before the agent’s behavior is explained. Our results not only suggest that mentalizing on robotic agents appears to be a two-step process, but that explaining behavior after the initial judgment phase appears to be quantifiably modified with increases in human-like appearance depending on the judged mental state. For example, more humanlike appearance may be necessary to make judgments about pain because of the belief that an agent would need something skin-like to feel pain if it tripped and fell on hard ground. Future studies should therefore investigate why different mental states need higher levels of human-like appearance to better understand the mechanisms involved in this secondary processing phase. In turn, robotic designers can incorporate minimal feature sets into their robotic designs saving both time and money while capitalizing on the natural processing observers engage in when trying to explain an agent’s behavior.

The current study provides strong evidence that physical appearance significantly affects perception of mental states in artificial agents and collaborating the two-step processing model outlined above by identifying different neural networks responsible for the qualitative and quantitative aspects observed will be important for future work. It is also important to recognize that other variables outside of appearance (i.e., behavior or beliefs about the agent) play an important role in HRI [3, 4, 7, 16–18] and might interact with or modulate appearance-based mind perception processes. In other words, seeing a robotic image, as opposed to interacting with the robot directly or having experience with its behavior, may elicit a mind perception process that is quantitatively and/or qualitatively different from the one that is currently described in the model. Thus, adding a behavioral component to the current model and testing its robustness will be important future steps for this line of research. Similarly, it needs to be investigated if this model holds across different rating scales of various anthropomorphic variables and how it may act upon other variables influencing mind attribution, such as dispositional (e.g., loneliness) and situational factors (e.g., availability of agent knowledge, sociality, and effectance motivation) in addition to appearance and behavior. Understanding how these different variables interact and affect an observer’s processing and judgment towards a non-human agent will provide further insights into human cognition and behavior.

## Conclusions

We have demonstrated that physical appearance plays a significant role in eliciting perceptions of intentionality across a wide variety of social and cognitive dimensions. Previous work has shown that adopting the intentional stance towards humans and non-human agents is highly flexible and the same stimulus can trigger both mechanistic and intentional interpretations depending on the beliefs participants have about an agent’s mind [6, 19]. However, by default, humans seem to be more reluctant to adopt the intentional stance towards artificial agents than to humans, which leads to reduced effectiveness in interactions with these agents (e.g., Wiese et al. [6]).

We attempted to address how attributing a mind and therefore taking an intentional stance can be manipulated by modifying physical appearance, one of the identified variables presented in our theoretical framework of mind attribution in HRI, and modeled its relationship mathematically. Our findings suggest that there is a qualitative split between agents considered ‘mindful’ and agents considered ‘mindless.’ It is only after a threshold, biased towards the human side of the spectrum and aligning with Looser & Wheatley’s [21] findings, that an agent based on appearance alone begins to be considered as having intentionality. Once this threshold is crossed the degree of human-likeness affecting mind attribution varies depending on the mental state being judged with some states requiring more human-like appearance than others. The observed categorical split followed by a quantitative change after the initial ‘mind’ threshold has been crossed suggests a two-step processing model where observers must first decide if an agent is intentional before judging various mental states. Consequently, if social roboticists wish to induce mind attribution in HRI, then they would be wise to include some degree of human-likeness into their designs. Specifically, the degree of human-likeness required within a design should be considered based on how much intentionality is needed for a given task and what types of interactions will occur. If a task does not require intentionality, then more human-like appearance may not be necessary, but it may have significant positive effects on HRI depending on what mental states designers want attributed to their mechanistic beings.

## Supporting Information

S1 Data Experiment 1 Raw Data. Lists the individual ratings given by each participant for each item in Experiment 1 along with demographics.
(CSV)

S2 Data Experiment 2 Raw Data. Lists the individual ratings given by each participant for each item in Experiment 2 along with demographics.
(CSV)

S1 Text Experiment 2 Item Number Key. Lists every item for all eight categories of Experiment 2 with item numbering corresponding to the item number seen in the S3 Data file.
(PDF)
