# Correction: Seeing Minds in Others – Can Agents with Robotic Appearance Have Human-Like Preferences?

**DOI:** 10.1371/journal.pone.0149766

**Published:** 2016-02-12

**Authors:** Molly C. Martini, Christian A. Gonzalez, Eva Wiese

The following information is missing from the Funding section: Publication of this article was funded in part by the George Mason University Libraries Open Access Publishing Fund.
