# Supplementary material for: Seeing Minds in Others – Can Agents with Robotic Appearance Have Human-Like Preferences?
Source: PLoS One. 2016 Jan 8;11(1):e0146310. doi: 10.1371/journal.pone.0146310 (PMC4706415; doi:10.1371/journal.pone.0146310)
Supplement: S1 Text — Lists every item for all eight categories of Experiment 2 with item numbering corresponding to the item number seen in the S3 Data file. (PDF) [file pone.0146310.s003.pdf]

Experiment 2 - Image Questions

* Instructions: You will now be shown a series of images representing different agents. Each image will be presented with a single question. For each question, please answer on a scale from 1 to 7 where 1 = *Definitely Not* and 7 = *Definitely*.

Agency

1. Do you think this agent is aware of its actions?

2. Do you think this agent can show goal directed behavior?

3. Do you think this agent plans out its own actions?

4. Do you think this agent can make it's own decisions?

5. Do you think this agent can make choices?

Animacy

6. Do you think this agent looks alive?

7. Do you think this agent has a mind?

8. Do you think this agent is conscious about its surroundings?

9. Do you think this agent can act on its own in the world?

10. Do you think this agent possesses self-reflection?

Theory of Mind

11. Do you think this agent can understand your intentions?

12. Do you think this agent can understand your emotions?

13. Do you think this agent can understand social behavior?

14. Do you think this agent can understand your preferences?

15. Do you think this agent can understand your actions?

Emotions

16. Do you think this agent can experience emotion?

17. Do you think this agent can experience happiness?

18. Do you think this agent can experience sadness?

19. Do you think this agent has feelings?

20. Do you think this agent can feel pain?

<u>Goals and preferences</u>

21. Do you think this agent has desires?

22. Do you think this agent has goals?

23. Do you think this agent has preferences?

24. Do you think this agent has plans for the future?

<u>Cognitive skills</u>

25. Do you think this agent is capable of complex behavior?

26. Do you think this agent can learn?

27. Do you think this agent has thoughts?

28. Do you think this agent can generate thoughts on its' own?

29. Do you think this agent strategizes to accomplish a given task?

<u>Social interactions/Communicative skills</u>

30. Do you think this agent would hang out with friends?

31. Do you think this agent is capable of taking care of another being?

32. Do you think this agent can react to the social signals of others?

33. Do you think this agent values the presence of others?

34. Do you think this agent could follow along with a conversation?

Sense of humor

35. Do you think this agent would understand humor?

36. Do you think this agent would like jokes?

37. Do you think this agent would understand irony, i.e., when the opposite of what is expected occurs?

38. Do you think this agent would understand sarcasm, i.e., the use of irony to mock or convey contempt?

39. Do you think this agent is able to tell jokes?
